# Therapeutic efficacy and pharmacological mechanism of Bailing capsule on chronic obstructive pulmonary disease: a meta-analysis and network pharmacology

**DOI:** 10.1080/13880209.2024.2415643

**Published:** 2024-10-26

**Authors:** Guanzhou Ma, Yang Jin

**Affiliations:** a Department of Respiratory and Critical Care Medicine, Hubei Province Clinical Research Center for Major Respiratory Diseases, Key Laboratory of Respiratory Diseases of National Health Commission, Union Hospital, Tongji Medical College, Huazhong University of Science and Technology, Wuhan, Hubei, China; b Hubei Province Engineering Research Center for Tumour-Targeted Biochemotherapy, MOE Key Laboratory of Biological Targeted Therapy, Union Hospital, Tongji Medical College, Huazhong University of Science and Technology, Wuhan, Hubei, China; c Hubei Province Key Laboratory of Biological Targeted Therapy, Union Hospital, Tongji Medical College, Huazhong University of Science and Technology, Wuhan, Hubei, China

**Keywords:** COPD, *Cordyceps sinensis*, traditional Chinese medicine, randomized controlled trials

## Abstract

**Context:**

Bailing capsule, derived from *Cordyceps sinensis* (Berk.) Sacc. (Clavicipitaceae), has shown potential in the treatment of chronic obstructive pulmonary disease (COPD), a prevalent respiratory disorder.

**Objective:**

This study elucidates the efficacy and mechanism of action of the use of Bailing capsules in the treatment of COPD using meta-analysis and network pharmacology.

**Materials and methods:**

A meta-analysis of randomized controlled trials (RCTs) was performed. The treatment group received Bailing capsules alongside standard therapy, while the control group received standard therapy or in combination with other traditional Chinese medicines. Efficacy outcomes included lung function, exercise tolerance, acute exacerbation risk, and quality of life. Network pharmacology and molecular docking identified key targets of Bailing capsules.

**Results:**

The meta-analysis of 27 RCTs showed significant improvements in the treatment group for forced expiratory volume in 1 s (FEV1), FEV1/FVC ratio, and the 6-min walk test (6MWT). Additionally, there was a marked reduction in acute COPD attacks and an improvement in quality of life. Meanwhile, network pharmacological analysis identified SRC, HIF1A, NFKB1, HDAC2, and PRKACA, as the potential core targets for Bailing capsules in the treatment of COPD.

**Discussion and conclusion:**

Bailing capsules have shown promising results in the treatment of stable COPD. Future studies should focus on large-scale, multicenter RCTs to confirm the long-term benefits and safety of Bailing capsules.

## Introduction

Chronic obstructive pulmonary disease (COPD) is thought to be a heterogeneous condition characterized by chronic respiratory symptoms such as dyspnea, cough, sputum production, frequent exacerbations, and progressive airflow obstruction. Pathologically, COPD encompasses bronchitis, obstructive bronchiolitis, emphysema, and pulmonary vascular changes. The inflammatory response varies by disease phenotype, primarily involving macrophages, neutrophils, and CD4 and CD8 T-lymphocytes (Cazzola et al. 2023; Venkatesan 2023). Although primarily a pulmonary disorder, COPD has also been shown to result in significant systemic effects in affected individuals. As one of the most prevalent respiratory diseases on a global scale, COPD has a high mortality rate and considerable socio-economic impact. COPD is the third leading cause of death globally, following ischaemic heart disease and stroke. It results in around 3 million deaths annually, accounting for approximately 4.72% of all deaths. Globally, the prevalence rate is around 11.8% among males and 8.5% among females (Papaioannou et al. 2024). In China, the prevalence of COPD in individuals over 40 is approximately 13.7%, noted for its high prevalence, disability, and mortality rates (Yin et al. 2022).

COPD profoundly affects the daily lives of patients. The effective management of COPD aims to reduce the frequency of exacerbations, slow the progression of lung function decline, and enhance quality of life, especially during the stable phases of the disease. Common pharmacological treatments include glucocorticoids, bronchodilators, antibiotics, and therapies for the symptomatic relief of cough and sputum, as well as for controlling infections or preventing complications (Roche 2018; Vogelmeier et al. 2020; Singh 2021). Despite these interventions, some patients may still suffer from severe complications such as respiratory failure, pulmonary heart disease, pulmonary encephalopathy, shock, and disseminated intravascular coagulation (DIC). Moreover, the progression of COPD can lead to comorbid conditions, including cardiovascular diseases, gastrointestinal dysfunction, and mental health issues, such as anxiety and depression. Today, the primary goals in the treatment of COPD involve symptom alleviation, improvements in lung function, enhancing patients’ quality of life, and minimizing or delaying acute exacerbations (Camac et al. 2021; Liu and Hui 2024).

Bailing capsule is a proprietary Chinese patent medicine composed of fermented *Cordyceps sinensis* (Berk.) Sacc. (Clavicipitaceae) powder (Cs-C-Q80), made from the dried powder of mycelium obtained through liquid deep fermentation, which contains key bioactive compounds such as cordycepin, adenosine, mannitol, and polysaccharides. It is traditionally utilized for its effects on tonifying the lungs and kidneys, alleviating coughs, and resolving phlegm. According to traditional Chinese medicine (TCM), Bailing capsule is notably effective in the treatment of prolonged coughs related to lung deficiency, wheezing from kidney deficiency, and labor-induced hemoptysis, which can significantly improve these symptoms. Recent research has also demonstrated its efficacy in managing COPD during the period of its clinical remission. Further basic studies indicate that the primary active compounds in Bailing capsule target specific molecules such as TP53, CTNNB1, TNF, and IL-6, thereby modulating the inflammatory response in COPD via the PI3K/Akt and cAMP signaling pathways (Yang et al. 2018; Yu et al. 2019; Lu et al. 2024).

Extensive research utilizing resources such as the TCM Systematic Pharmacology Database (TCMSP) and the Swiss Target Prediction Database has identified the components and targets of Bailing capsule. The related targets of COPD were analyzed by using GeneCards, DrugBank, the Online Mendelian Inheritance in Man (OMIM), and the Therapeutic Target Database (TTD). The protein–protein interaction network was constructed by using STRING and visualized. Molecular docking was validated through AutoDock Vina. Network pharmacology and molecular docking studies help to establish a robust framework for understanding how to modulate COPD with Bailing capsule. This research aligns with the current COPD guidelines, emphasizing improvements in lung function, exercise tolerance, risk assessment, and quality of life for stable COPD patients. Additionally, it explores the pharmacological network, potential targets, and molecular interactions of Bailing capsule, and provides essential insights for its clinical use in the stable phase of COPD. These insights contribute to controlling and improving symptoms and quality of life, potentially broadening the clinical benefits of Bailing capsules for more patients of COPD.

## Materials and methods

### Search strategy

To enhance our understanding of the clinical utility and effectiveness of Bailing capsules during the treatment of the stable phase of COPD, we conducted a comprehensive literature search across various databases, including CNKI, Wanfang, VIP, China CBM, SinoMed, PubMed, Embase, MEDLINE, the Cochrane Library, and Web of Science. Our search strategy utilized specific keywords associated with the treatment and efficacy of COPD, such as ‘Cordyceps’, ‘Bailing capsule’, ‘chronic obstructive pulmonary disease’, ‘COPD’, ‘COAD’ (chronic obstructive airway disease), ‘chronic obstructive lung disease’, and ‘chronic airflow obstruction’. Keywords within similar categories were linked using ‘or’, while the relationship between therapeutic interventions and the disease state was denoted by ‘and’.

### Inclusion and exclusion criteria

This study reviewed RCTs that included subjects diagnosed with stable COPD per recognized guidelines. Participants of any age and sex were eligible, regardless of the duration of COPD. The control group adopted standard treatments, either conventional Western medicine alone or combined with traditional Chinese medicine. Conversely, the experimental group just administered Bailing capsules along with conventional standard treatments. Other standard treatments included oxygen therapy, nebulized antispasmodics, anti-asthmatics, anti-infectives, rehabilitation, and nutritional support. Trials that failed to meet these criteria were excluded. Additionally, patients suffering from recent respiratory failure or severe comorbidities were not included in the study as well.

### Literature selection and data extraction

Literature selection and data extraction for this study were carried out by two independent researchers, who strictly followed predefined screening criteria. Initially, they reviewed the titles and abstracts of articles, progressing to detailed examinations of the full texts as per these criteria. This methodical approach could ensure a thorough and impartial review of all studies that met the inclusion standards.

The researchers compiled the data that included participant age, gender, sample size, the details of both intervention and control groups, treatment duration, efficacy outcomes, prognosis, and any noted adverse effects. To maintain accuracy and consistency, both researchers cross-verified the selected studies and extracted the data. When there is a disagreement in opinions, issues are resolved through consultation with a third-party expert.

### Risk of bias assessment

The risk of bias was evaluated via the assessment tool recommended by the Cochrane Handbook (Higgins et al. 2023) in the study. This tool examines seven key areas: the method of generating randomized sequences, concealment of allocation, blinding of patients and trial personnel, blinding of outcome assessors, completeness of outcome data, selective outcome reporting, and the other potential sources of bias.

Each study was rigorously appraised for its quality and content as well. Based on the relevant evaluation and analysis, all the studies were categorized as having a ‘low risk’, ‘high risk’, or ‘unclear’ risk of bias. This systematic assessment ensured that our analysis was grounded in studies with the most reliable and unbiased data. To assess clinical heterogeneity, only studies with similar conditions and treatments were compared for clinically useful results.

### Network pharmacology analysis of Bailing capsules for COPD treatment

#### Active ingredients and targets identification in Bailing capsules

Active ingredients of Bailing capsules were identified using the TCMSP database and other relevant databases, employing oral bioavailability (OB) ≥ 30% and drug-likeness (DL) ≥ 0.18 as the screening criteria. For instances where the Herbal Historia (Herb) database (http://herb.ac.cn) was used, five principles of Lipinski were applied to guide the screening of active ingredients. The isomeric SMILES numbers of potential compounds were obtained from the PubChem database using the PubChem CIDs. If no PubChem CID was available, these numbers were calculated from molecular images in TCMSP through the Structure Calculation Station (http://www.vcclab.org/web/alogps/). These SMILES numbers were then input into the SwissTargetPrediction and TargetNet databases to predict and identify compound targets. Finally, the drug-component-target network was constructed by using Cytoscape software based on these identified targets.

#### Disease target screening

Disease-related targets for COPD were identified by using databases such as GeneCards and DisGeNET. These targets were de-duplicated and normalized *via* the Uniprot database.

#### Potential target protein–protein interaction networks

The interaction between disease-related and TCM-related targets was mapped using Venn, identifying intersection targets presented as Venn diagrams. These intersected targets were then input into the String database (with the species set as ‘Homo sapiens’ and other parameters left as default) to generate a protein–protein interaction network. This network was subsequently imported into Cytoscape, where Degree (DC), Betweenness (BC), and Closeness (CC) values were calculated using CytoNCA to identify the key targets.

#### Drug–disease target outcome prediction

‘Network.xlsx’ and ‘type.xlsx’ files, containing drug-disease-target data, were created and imported into Cytoscape 3.8.2 for mapping and further analysis.

#### Molecular docking study in cyber pharmacology of Bailing capsules

The molecular docking study aimed to predict and elucidate the binding interactions between small molecules (ligands) and the target proteins. Semi-flexible docking was employed, where the target proteins remained rigid while the ligands adapted their conformation. Based on prior analysis, the top five critical targets were selected for semi-flexible docking with the main components of Bailing capsules. Then, the binding interactions were also analyzed in terms of binding energies (Affinity).

### Statistical analysis

Statistical analyses were conducted using RevMan (version 5.4) and Stata (version 16.0) software to facilitate the construction of a comprehensive treatment strategy network. The analyses included both dichotomous and continuous variables. Dichotomous variables were expressed as odds ratios (ORs), while continuous variables were represented by mean difference (MD) or standardized mean difference (SMD). All interval estimates were accompanied by 95% confidence intervals (CI). The difference between the two groups was deemed statistically significant if a 95% confidence interval for MD or SMD did not encompass zero. Heterogeneity was assessed by *I*^2^ statistics and explored through sensitivity analysis. Using Egger regression tests to assess possible publication bias.

Additionally, Stata software was crucial in processing outcome data. It was used to develop a network diagram specifically for Bailing capsule intervention, comparing the same outcome index across studies. This approach effectively illustrated the treatment effects and differences associated with Bailing capsule.

## Results

### Literature search and characteristics of the included studies

**Figure 1.**
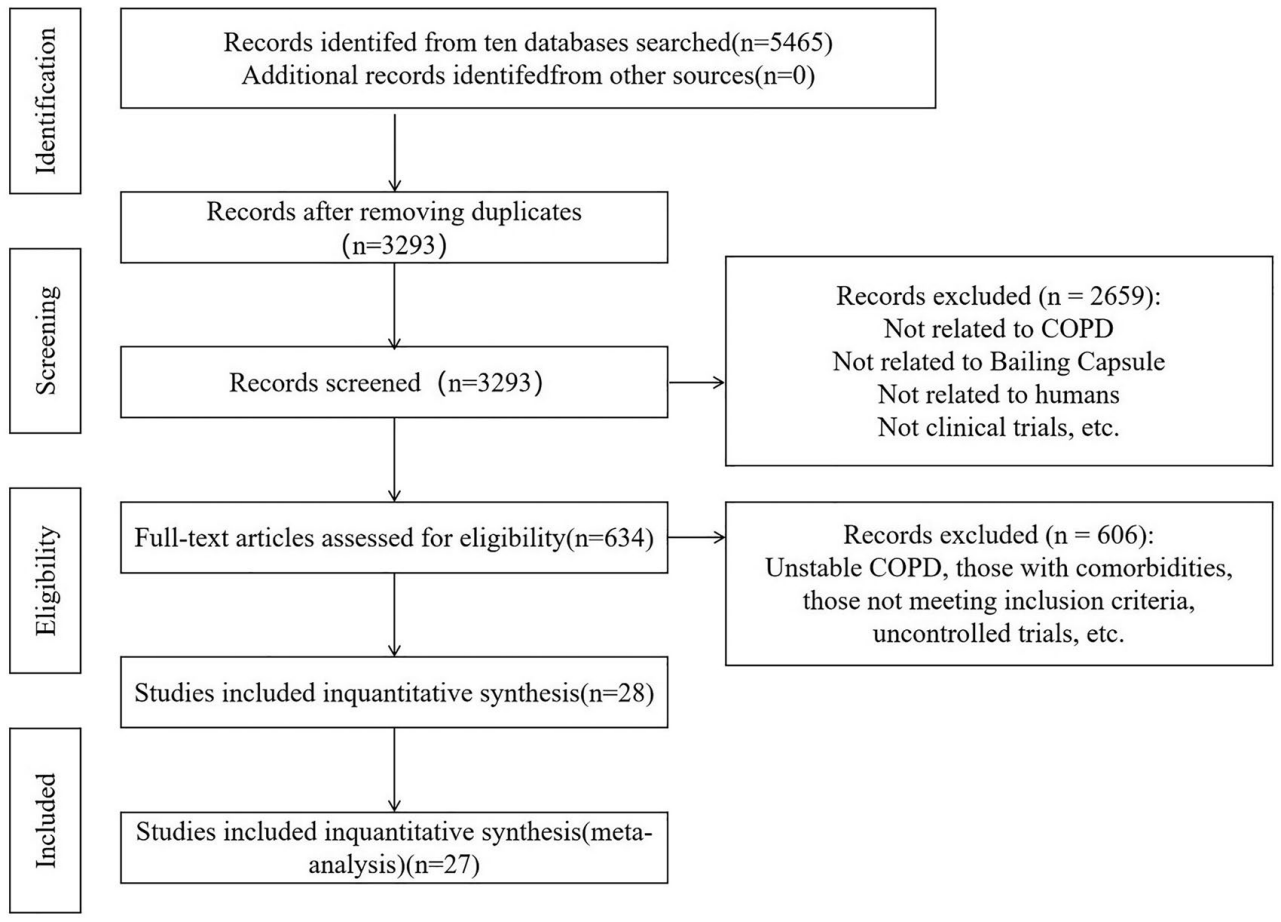
PRISMA study flowchart of search results

We identified a total of 5,465 documents, of which 3,293 remained after removing duplicates. Following the preliminary screening, 2,659 documents were excluded for reasons such as not being about COPD and Bailing capsule, being non-human studies, or not being clinical trials. Further review of the full texts of 634 documents led to the exclusion of 606 papers due to reasons, including unstable COPD, comorbidities, non-compliance with inclusion criteria, or uncontrolled studies. Ultimately, 28 studies were chosen for quantitative synthesis, with 27 of these studies contributing to the meta-analysis. This selection process is illustrated in the PRISMA flowchart (Figure 1), for ensuring the rigor and relevance of the research.

These trials, all conducted in China, involved a total of 2,622 participants who varied in age and gender, and with studying treatment durations ranging from 15 days to 1 year (Table 1). The changes in pulmonary function were observed in 19 studies, specifically forced expiratory volume in 1 s (FEV1) (mean ± SE), as the representative indicator of lung function, as detailed in numerous sources (Huang et al. 2005; Mao 2009; Zeng et al. 2011; Xu and Xu 2015; Huang 2018; Li et al. 2018; Liu and Liu 2018; She 2018; Wang 2018; Zhang et al. 2018; Li 2019; Wang and Wang 2019; Yang 2019; Guan 2020; Zhang 2020; Hao et al. 2021; Wang et al. 2021; Zheng and Li 2021; Jiang 2022). Additionally, the parameter of the FEV1/FVC ratio was also measured in the 21 studies mentioned above (Huang et al. 2005; Mao 2009; Xu and Xu 2015; Huang 2018; Li et al. 2018; Liu and Liu 2018; Pang 2018; She 2018; Wang 2018; Zhang et al. 2018; Li 2019; Li et al. 2019; Wang and Wang 2019; Yang 2019; Guan 2020; Zhang 2020; Hao et al. 2021; Wang et al. 2021; Xing et al. 2021; Zheng and Li 2021; Jiang 2022). Four studies provided details on the Risk assessment for acute exacerbation of COPD within one year (mean ± SE) (Huang et al. 2005; Mao 2009; Li et al. 2019; Zhang 2020). Three studies provided details on the 6MWT (mean ± SE) (Mao 2009; Liu and Liu 2018; Xu et al. 2019). Moreover, the patients’ outcomes were assessed by using the St. George’s Respiratory Questionnaire (SGRQ) (mean ± SE) in 7 studies (Huang et al. 2005; Xing et al. 2013; Xu and Xu 2015; Pang 2018; Li 2019; Liu 2019; Wang et al. 2021).

**Table 1.** Basic characteristics of the included studies.

Inclusion of studies	Group	Sample size	Male/female	Treatment measures	Treatment time	Outcome indicators
Huang D et al. (2005)	Treatment	32	21/11	Standard WM + Yupingfeng granules, Bailing capsules	3 months	①②③④⑤
	Control	31	23/8	Standard WM	3 months	①②③④⑤
Mao (2009)	Treatment	58	42/16	Standard WM + Bailing capsules	3 months	①②⑥
	Control	50	39/ 11	Standard WM	3 months	①②⑥
Zeng et al. (2011)	Treatment	42		Standard WM + Bailing capsules	9 months	①②⑦
	Control	48		Standard WM	9 months	①②⑦
Xing et al. (2013)	Treatment	37	20/17	Standard WM + Bailing capsules	12 weeks	③
	Control	38	21/17	Standard WM	12 weeks	③
Xu and Xu (2015)	Treatment	50	30/20	Standard WM + Bailing capsules, acupoint therapy	12 weeks	①③⑦⑧
	Control	50	32/18	Standard WM	12 weeks	①③⑦⑧
Yuan et al. (2018)	Treatment	50	32/18	Standard WM + Bailing capsules	12 weeks	⑥
	Control	50	36/14	Standard WM	12 weeks	⑥
Liu and Liu (2018)	Treatment	59	29/30	Standard WM + Bailing capsules, respiratory rehabilitation	1 year	①⑥⑨⑩
	Control	58	27/31	Standard WM	1 year	①⑥⑨⑩
Cai (2018)	Treatment	84	51/33	Standard WM + Bailing capsules	3 months	⑪⑫
	Control	83	49/34	Standard WM	3 months	⑪⑫
Li et al. (2018)	Treatment	30	13/17	Standard WM + Bailing capsules	3 months	①⑬⑭
	Control	30	14/16	Standard WM	3 months	①⑬⑭
Zhang et al. (2018)	Treatment	52	31/21	Standard WM + Bailing capsules	12 weeks	①⑮
	Control	52	29/23	Standard WM	12 weeks	①⑮
Pang 2018	Treatment	40	23/17	Standard WM + Bailing capsules	6 months	①③
	Control	40	24/16	Standard WM	6 months	①③
She (2018)	Treatment	61	32/29	Standard WM + Bailing capsules	8 weeks	①⑪⑯
	Control	61	38/23	Standard WM	8 weeks	①⑪⑯
Huang (2018)	Treatment	41	24/17	Standard WM + Bailing capsules	2 months	①⑰
	Control	41	23/18	Standard WM	2 months	①⑰
Wang (2018)	Treatment	60	33/27	Standard WM + Bailing capsules	12 weeks	①⑯
	Control	60	24/26	Standard WM	12 weeks	①⑯
Liu (2019)	Treatment	40	20/20	Standard WM + Bailing capsules	12 weeks	③⑱
	Control	40	18/22	Standard WM	12 weeks	③⑱
Li et al. (2019)	Treatment	31	27/4	Standard WM + Bailing capsules	8 weeks	①②⑲⑳㉑
	Control	31	25/6	Standard WM	8 weeks	①②⑲⑳㉑
Wang and Wang (2019)	Treatment	40	22/18	Standard WM + Bailing capsules	8 weeks	①㉒㉒
	Control	40	23/17	Standard WM	8 weeks	①㉒㉒
Li (2019)	Treatment	31	17/14	Standard WM + Bailing capsules	8 weeks	①③
	Control	29	15/14	Standard WM	8 weeks	①③
Yang (2019)	Treatment	56	32/24	Standard WM + Bailing capsules	2 months	①⑧㉔
	Control	56	34/22	Standard WM	2 months	①⑧㉔
Xu et al. (2019)	Treatment	60	36/24	Standard WM + Bailing capsules	1 year	①⑥⑮
	Control	60	32/28	Standard WM	1 year	①⑥⑮
Guan (2020)	Treatment	72	41/31	Standard WM + Bailing capsules	2 months	①⑧
	Control	72	44/28	Standard WM	2 months	①⑧
Zhang (2020)	Treatment	20	11/9	Standard WM + Bailing capsules	15 days	①②⑯
	Control	20	12/8	Standard WM	15 days	①②⑯
Hao et al. (2021)	Treatment	55	30/25	Standard WM + Bailing capsules	2 months	①㉒
	Control	55	32/23	Standard WM	2 months	①㉒
Wang et al. (2021)	Treatment	100	67/33	Standard WM + Bailing capsules	8 weeks	①③⑦㉑
	Control	100	65/35	Standard WM	8 weeks	①③⑦㉑
Xing et al. (2021)	Treatment	32	25/7	Standard WM + Bailing capsules	8 weeks	①②㉕
	Control	33	26/7	Standard WM	8 weeks	①②㉕
Zheng and Li (2021)	Treatment	45	29/16	Standard WM + Bailing capsules	3 months	①㉖
	Control	42	23/19	Standard WM	3 months	①㉖
Jiang (2022)	Treatment	37	26/11	Standard WM + Bailing capsules	8 weeks	①⑧⑯㉗
	Control	37	23/14	Standard WM	8 weeks	①⑧⑯㉗

WM: Western medicine.

① Lung Function Evaluation: Forced Vital Capacity (FVC), Forced Expiratory Volume in one second (FEV1), and FEV1/FVC% ratio.

② 6-Minute Walk Distance (6MWD).

③ Quality of Life: Assessed using the St. George’s Respiratory Questionnaire (SGRQ) for respiratory diseases.

④ Acute Exacerbation: Number and duration of acute exacerbations within 6 months after treatment.

⑤ General Safety Indicators: Blood, urine, stool routine tests, ALT, AST, BUN, and Cr. These indicators, except for the number and duration of acute exacerbations, are measured before and after treatment.

⑥ 1-Year Follow-Up: Number of acute exacerbations and stable days within one year.

⑦ BODE Index: Uses the BODE scoring system proposed by Celli et al. Body Mass Index (B) is weight/height². Functional dyspnea score (D) is based on the modified Medical Research Council (MMRC) grading system:.

⇁0: No noticeable dyspnea except during strenuous activities.

⇁1: Shortness of breath when walking quickly or on a slight incline.

⇁2: Walks slower than people of the same age due to shortness of breath or needs to stop while walking at own pace.

⇁3: Stops for breath after walking 100 meters or a few minutes on flat ground.

⇁4: Too breathless to leave the house or becomes short of breath while dressing.

⑧ Lung and Kidney Qi Deficiency Score: Based on "Guidelines for Clinical Research of New Chinese Medicine Drugs (Trial)." Scores range from 0 to 3, representing none, mild, moderate, and severe symptoms.

⑨ Quality of Life Pre and Post Treatment: Evaluates symptoms, activities, and the impact of COPD on social and psychological activities. Higher scores indicate greater impact.

⑩ Hamilton Anxiety Rating Scale (HAMA) and Hamilton Depression Scale (HAMD): Higher HAMA scores indicate severe anxiety. Higher HAMD scores indicate severe depression.

⑪ Resolution Time of Symptoms: Time to resolve symptoms like dyspnea, coughing, wheezing, and chest tightness.

⑫ Serum Th17/Treg Levels: Measured before treatment and three months after treatment.

⑬ Nutritional Index Changes: Includes BMI, hemoglobin (Hb), serum albumin (ALB), prealbumin (Palb), and triceps skinfold thickness (TSF).

⑭ Primary Symptom Score Changes: Includes coughing, expectoration, and wheezing, rated 0-3 (higher scores indicate more severe symptoms). Efficacy evaluation:.

⇁Significant improvement: >50% improvement in clinical symptom scores.

⇁Effective: 30–50% improvement.

⇁Ineffective: <30% improvement.

⑮ CD3+/CD4+ T-Cell Measurement: T-cell measurements before and 12 weeks after treatment using flow cytometry.

⑯ Arterial Blood Gas Indices: PaO2 and PaCO2 measured before and after treatment.

⑰ Serum MCP-1 Levels: Measured before and after treatment.

⑱ Activities of Daily Living (ADL): Higher ADL scores indicate greater ability.

⑲ Borg Dyspnea Scale: Higher Borg scores indicate more severe breathlessness.

⑳ Cognitive Function Scores: Based on Montreal Cognitive Assessment (MoCA) and Mini-Mental State Examination (MMSE).

㉑ Serum 8-Hydroxy-2’-Deoxyguanosine (8-OHdG) and Monocyte Chemoattractant Protein-1 (MCP-1) Levels: Blood samples collected in the morning after fasting.

㉒ Small Airway Function: Maximal expiratory flow at 50% (MEF50) and 25-75% (MEF25-75) of Forced Vital Capacity, and MEF25.

㉒ Airway Remodeling Molecular Factors: TGF-β1, NOX4, NF-κB, and MMP-9 measured before and eight weeks after treatment.

㉔ Serum IL-8, TNF-α, IgM, IgA, and IgG Levels: Measured before and two months after treatment.

㉕ Modified British Dyspnea Scale (mMRC): Patients complete this scale independently, which is collected immediately after filling.

㉖ Serum Cytokine Measurement: IL-1β, IL-6, IL-8 levels measured using ELISA.

㉗ Oxidative Stress Indices: Serum Superoxide Dismutase (SOD) and Malondialdehyde (MDA) levels before and after treatment.

### Risk of bias

**Figure 2.**
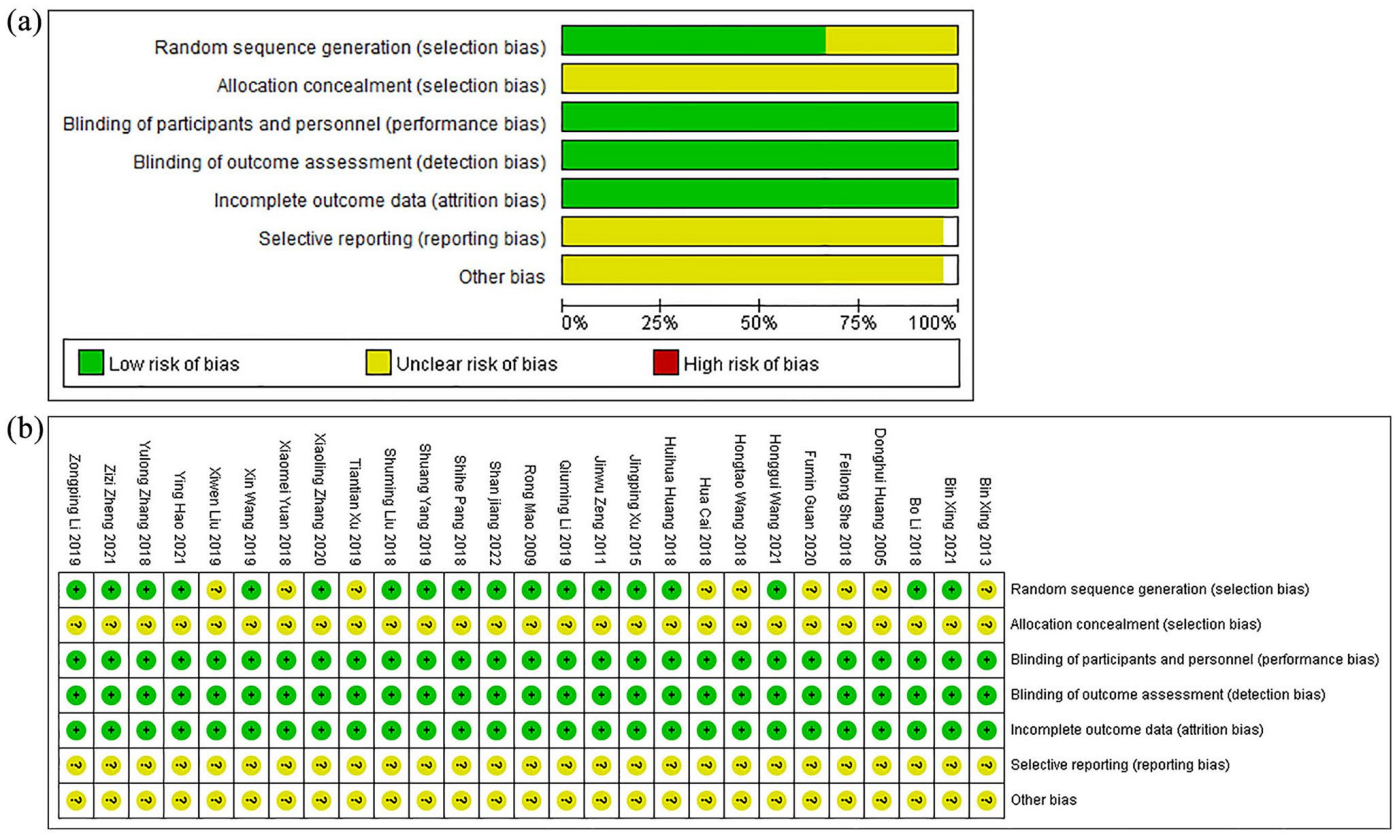
Assessment of the risk of bias. (a) Risk of bias graph of included literature. (b) Risk of bias summary of included literature.

Among the 27 RCTs, 18 of these studies explicitly stated that because the appropriate allocation methods, such as random number tables or random cards, were used in the research, all the outcomes of studies may generate a low risk of bias. However, the remaining studies only mentioned ‘random grouping’ without providing specific methodology in detail, resulting in their classification as having an unclear risk of bias. At the same time, none of the studies detailed methods for concealing the allocation scheme, which can lead to an unclear risk of bias assessment in this aspect as well. Since none of the 27 RCTs implemented blinding, the potential bias for the lack of blinding was primarily evaluated based on the impact of the results depending on subjective judgment. As the selected outcome measures in these studies were not susceptible to subjective influence, they were all rated as having a low risk of bias. Because the methods and results sections in 27 RCTs provided consistent data, the completeness of the outcome data was assessed as having a low risk of bias. Owing to the shortage of relevant descriptions across all 27 RCTs, the risk of bias of the selective outcome reporting and other potential sources of bias were assessed as unclear. The methodological quality of the studies included is displayed in Figure 2(a and b).

### Indicators associated with lung function

#### FEV1

**Figure 3.**
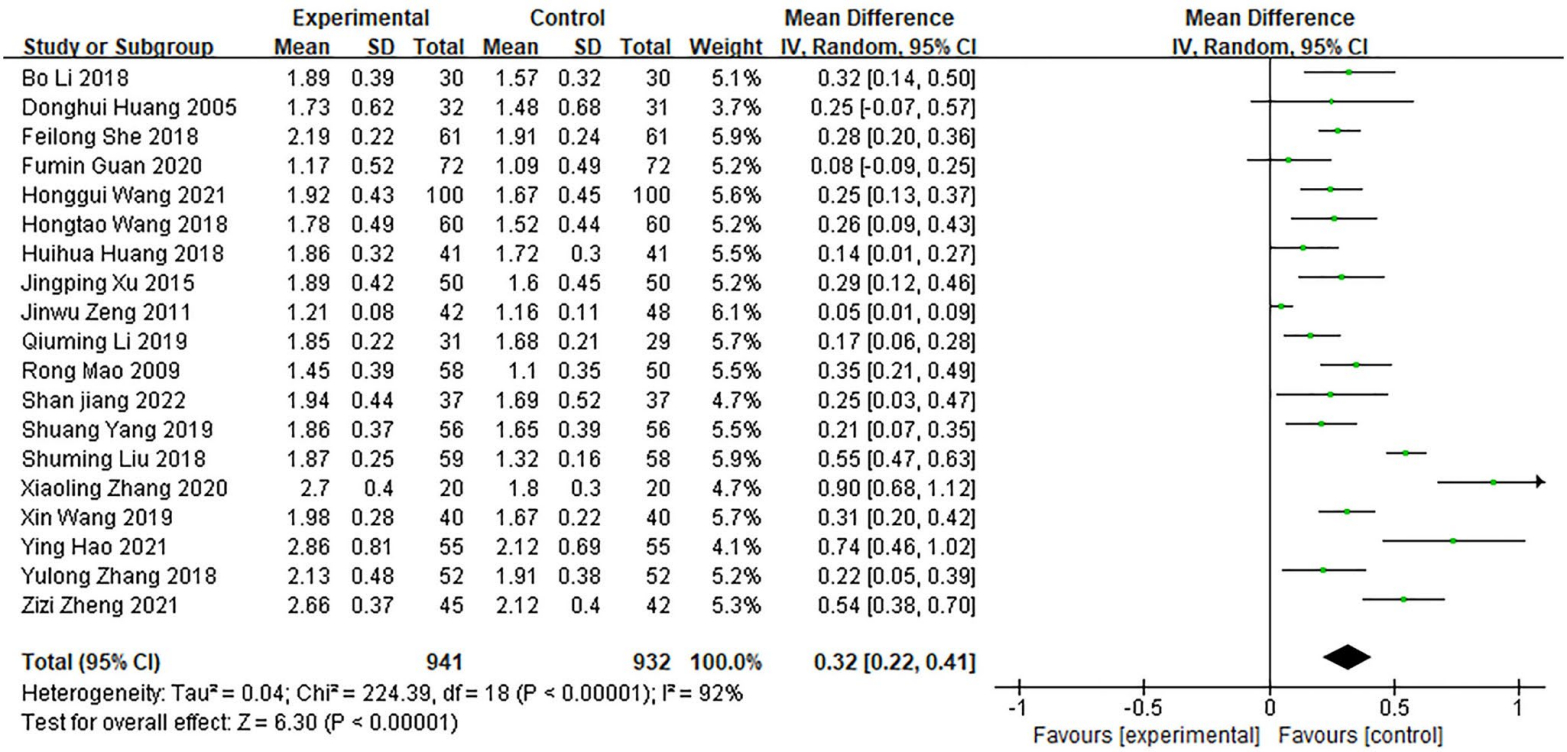
Forest plot of meta-analysis of FEV1 after treatment.

FEV1 assessment included 19 studies with a total of 1,873 patients, including 941 in the experimental group and 932 in the control group (Huang et al. 2005; Mao 2009; Zeng et al. 2011; Xu and Xu 2015; Huang 2018; Li et al. 2018; Liu and Liu 2018; She 2018; Wang 2018; Zhang et al. 2018; Li 2019; Wang and Wang 2019; Yang 2019; Guan 2020; Zhang 2020; Hao et al. 2021; Wang et al. 2021; Zheng and Li 2021; Jiang 2022). A significant heterogeneity was observed (*p*** ***<*** **0.00001, *I*^2^ = 92%). Using a random effects model, the indicator of FEV1 of patients after treatment of Bailing capsule and the conventional treatment was found to be higher than the conventional treatment alone [MD = 0.32, 95% CI (0.22,0.41), *p*** ***<*** **0.00001] (Figure 3). Sensitivity analysis, employing a literature exclusion method, did not significantly alter the heterogeneity, which demonstrated the robustness of the results.

#### FEV1/fvc

**Figure 4.**
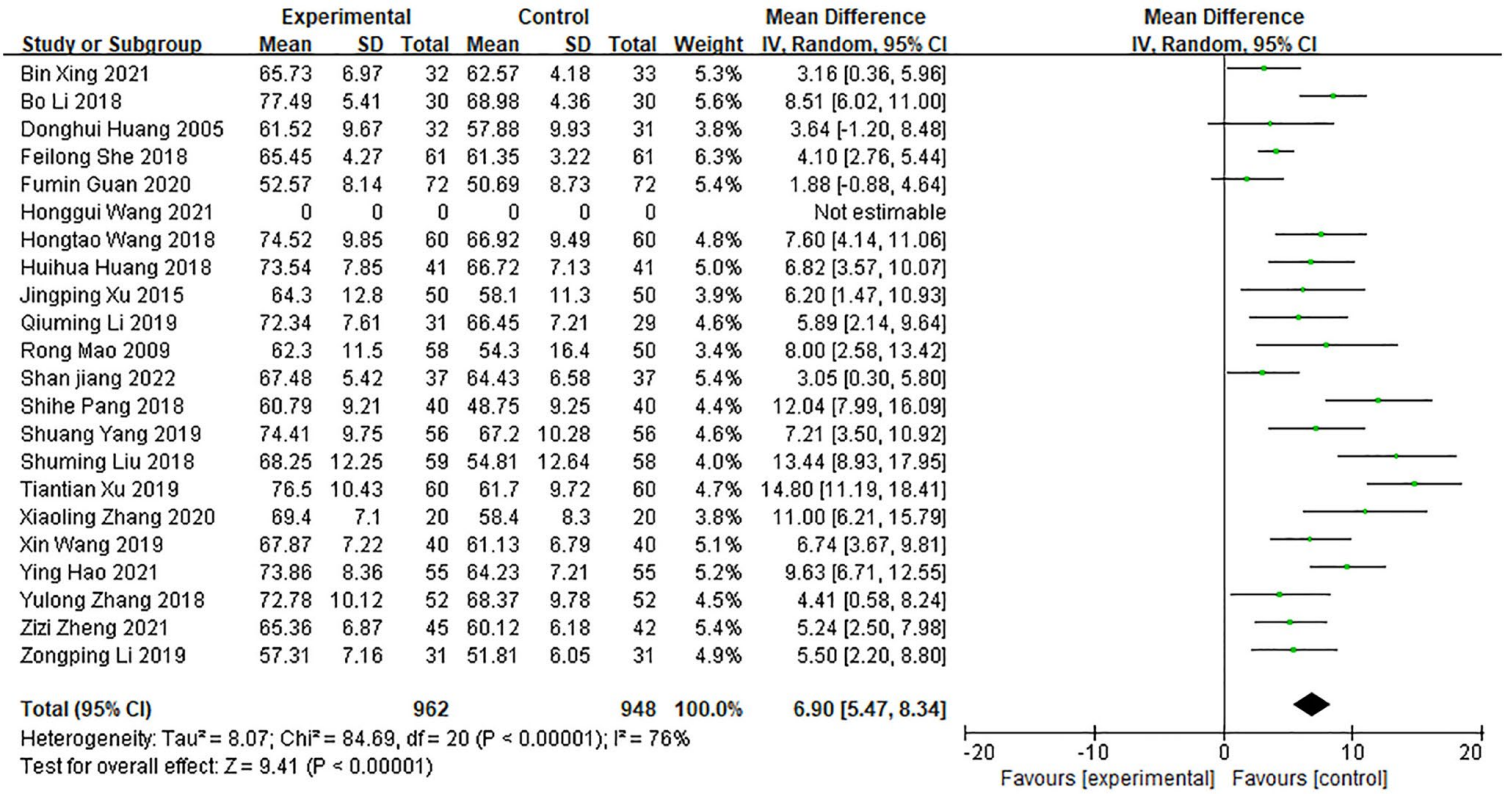
Meta-analyzed Forest plot of FEV1/FVC after treatment.

FEV1/FVC assessment included 21 studies with a total of 1,910 patients, of which 962 in the experimental group and 948 in the control group, were carefully analyzed (Huang et al. 2005; Mao 2009; Xu and Xu 2015; Huang 2018; Li et al. 2018; Liu and Liu 2018; Pang 2018; She 2018; Wang 2018; Zhang et al. 2018; Li 2019; Li et al. 2019; Wang and Wang 2019; Yang 2019; Guan 2020; Zhang 2020; Hao et al. 2021; Wang et al. 2021; Xing et al. 2021; Zheng and Li 2021; Jiang 2022). It showed a significant heterogeneity (*p*** ***<*** **0.00001, *I*^2^ = 76%), which warranted the feasibility of a random effects model for meta-analysis (Figure 4). The FEV1/FVC index in patients treated with Bailing capsule and conventional treatment was higher compared to conventional treatment alone [MD = 6.9, 95% CI (5.47, 8.34), *p*** ***<*** **0.00001]. Sensitivity analysis *via* literature exclusion showed a decrease in heterogeneity among the remaining studies (*I*^2^ = 47%, *p*** ***=*** **0.02), without any significant change in results when analyzed by using a fixed-effects model.

### Risk assessment for acute exacerbation of COPD within one year

**Figure 5.**
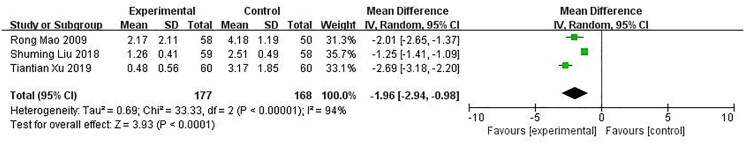
Forest plot of the risk analysis of the acute exacerbation of COPD within 1 year after treatment.

The risk assessment for acute exacerbation of COPD within one year included three studies with a total of 345 patients (177 in the experimental group and 168 in the control group) included in this analysis (Mao 2009; Liu and Liu 2018; Xu et al. 2019). Significant heterogeneity was detected (*p*** ***<*** **0.00001, *I*^2^ = 94%), leading to the use of a random effects model for meta-analysis (Figure 5). The analysis indicated that the number of acute exacerbations of COPD within one year was significantly lower in patients treated with Bailing capsule and the conventional treatment compared to the conventional treatment alone [MD = −1.96, 95% CI (−2.94, −0.98), *p*** ***<*** **0.00001]. Sensitivity analysis using a literature exclusion method showed a reduction in heterogeneity among the studies (*I*^2^ = 29%, *p*** ***=*** **0.24), with consistent results when analyzed using a fixed effects model.

#### Motor function assessment (6MWT)

**Figure 6.**
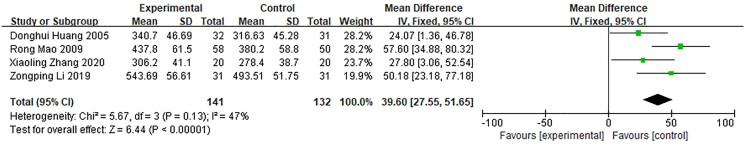
Forest plot of meta-analysis of 6MWT after treatment.

6MWT assessment included four studies with a total of 273 patients, comprising 141 patients in the experimental group and 132 in the control group (Huang D et al. 2005; Mao 2009; Li et al. 2019; Zhang 2020). The heterogeneity test yielded no significant heterogeneity (*p*** ***=*** **0.13, *I*^2^ = 47%), allowing for a meta-analysis using a fixed effects model (Figure 6). The results demonstrated a significant improvement in walking ability and performance measured by the 6MWT for the patients in the experimental group, compared to the control group [MD = 39.6, 95% CI (27.55, 51.65), *p*** ***<*** **0.00001].

#### SGRQ scores of quality of life

**Figure 7.**
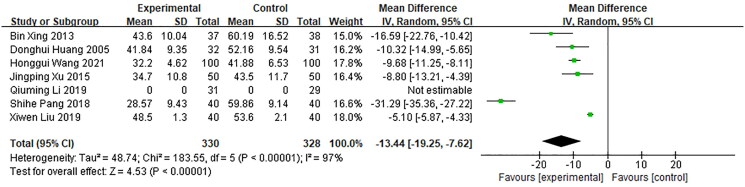
Forest plot of meta-analysis of SGRQ after treatment

SGRQ scores of quality of life were assessed in seven studies, including 658 patients (330 in the experimental group and 328 in the control group) (Huang et al. 2005; Xing et al. 2013; Xu and Xu 2015; Pang 2018; Li 2019; Liu 2019; Wang et al. 2021). The heterogeneity test results (*p*** ***<*** **0.00001, *I*^2^ = 97%) indicated extremely high heterogeneity, prompting the use of a random effects model for the meta-analysis (Figure 7). The analysis revealed that the treatment of Bailing capsules combined with the conventional treatment can improve the patient’s quality of life [MD = −13.44, 95% CI (−19.25, −7.62), *p*** ***<*** **0.00001]. A sensitivity analysis using the literature exclusion method indicated a significant reduction in heterogeneity (*I*^2^ = 38%, *p*** ***=*** **0.18). The results were consistent with the findings when analyzed using a fixed effects model.

### Publication bias

**Figure 8.**
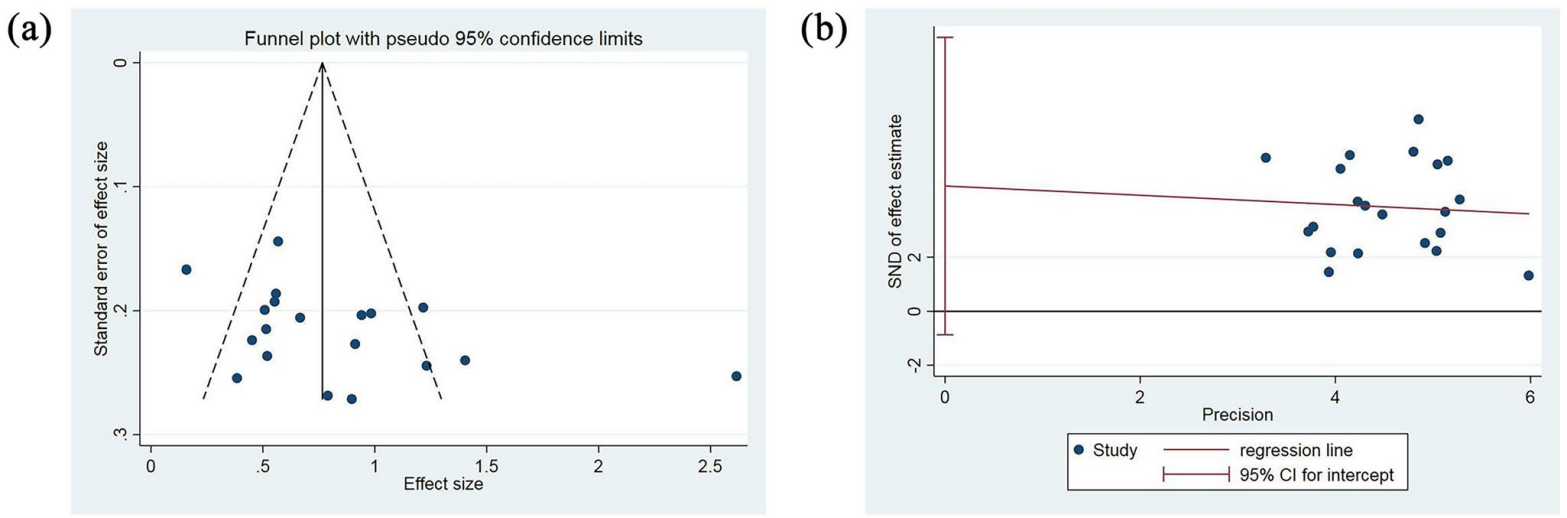
Funnel plot analysis. (a) Funnel plot of data bias analysis. (b) Funnel plot generated by egger’s linear regression method for offset detection

Publication bias of FEV1 and FEV1/FVC were assessed using the funnel plot as depicted in Figure 8(a and b). Because the number of included studies that reported 6MWT, Risk assessment for acute exacerbation of COPD within one year, and SGRQ scores were <10, we did not assess the publication bias for them.

A funnel plot is a scatterplot that displays the intervention effect estimates from individual studies against their respective sample size or precision. This approach is instrumental in identifying potential publication bias. In the plot, the horizontal axis represents the effect estimates of individual studies, expressed as odds ratios (ORs), while the vertical axis denotes the standard error related to the sample size during the study. The scatter points in this funnel plot are primarily concentrated at the bottom due to the larger standard errors associated with smaller study sizes. Most scatter points are distributed around the central ORs however, some points fall outside the confidence interval, suggesting a possibility of substantial bias (Figure 8(a)).

Egger’s linear regression method was also applied to measure the symmetry of the inverted funnel plot, using the natural logarithm of the odds ratio. This method is highly effective for both continuous and dichotomous data, allowing the assessment of the symmetry of data adoption through the regression equation intercept. The 95% confidence interval of the intercept indicates the degree of symmetry. An intercept close to zero suggests minimal bias in the adoption data, whereas a value far from zero will indicate a potential skew in the analyzed data (Figure 8(b)).

### Network pharmacology and molecular docking studies

#### Core targets and network interactions

**Figure 9.**
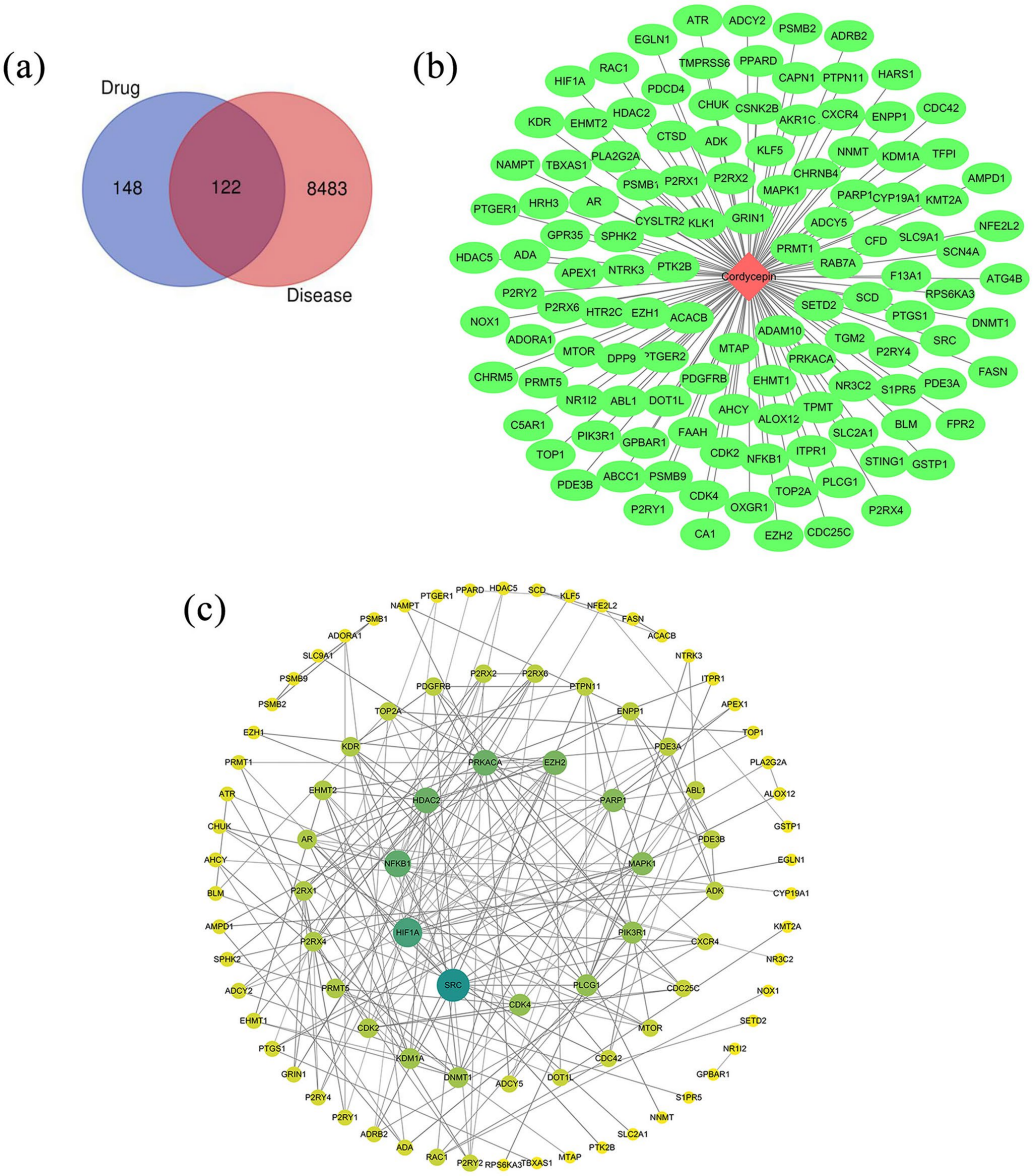
Network pharmacology analysis of Bailing capsule: Venn diagram, protein–protein interaction network, and targets associated with Bailing capsule. (a) Veen diagram. (b) Protein–protein interaction network. (c) Network analysis showing targets associated with Bailing capsule

Shared drug and disease targets (122) were identified by Venn diagram analysis. These targets were imported into the STRING database (https://string-db.org/) for protein-protein interaction prediction (Figure 9(a)). Setting the species to Homo sapiens, the network file was saved in TSV format and subsequently imported into Cytoscape 3.8.2 for visualization and topological analysis. The resulting network comprised 90 nodes and 202 edges, with targets displayed in varying sizes and colors according to their degree values. The combined score values could determine the edge thicknesses. The identified core targets include SRC, HIF1A, NFKB1, HDAC2, and PRKACA (Figure 9(b)).

#### Drug–disease target prediction results

**Figure 10.**
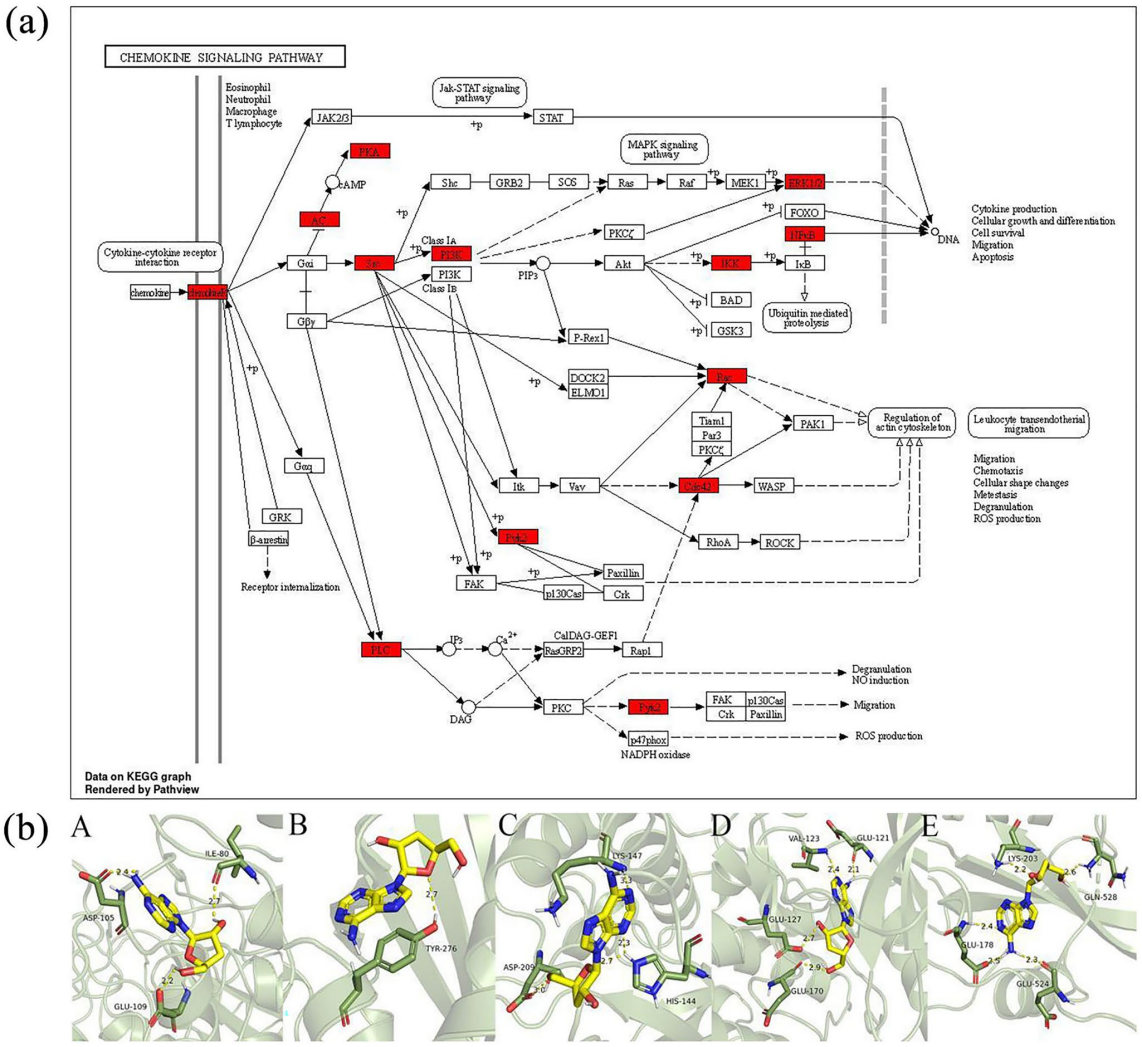
Potential mechanisms of action for Bailing capsule: Chemokine signaling pathways and molecular targets of cordycepin. (a) Chemokines and molecular signaling pathways potentially involved in Bailing capsule’s action. (b) Interaction patterns of cordycepin with key targets (A. Mode of the interaction of cordycepin with HDAC2. B. Mode of the interaction of cordycepin with HIF1A. C. Mode of the interaction of cordycepin with NFKB1. D. Mode of the interaction of cordycepin with PRKACA. E. Mode of the interaction of cordycepin with SRC.).

In this analysis, targets related to COPD and those associated with Bailing capsule were mapped against each other. This mapping process yielded a total of 122 intersecting drug-disease genes. Utilizing this drug-disease-target data, ‘network.xlsx’ and ‘type.xlsx’ files were created and subsequently imported into Cytoscape 3.8.2 for network construction. The resulting network comprised 123 nodes and 122 edges. The target analysis connected with the effects of Bailing capsule revealed the interaction with multiple targets. Prominent among these targets were genes such as CXCR4, PDGFRB, and PARP1 (Figure 9(c)). The analysis further suggested that the mechanism might involve interactions with chemokines, tyrosine kinase receptors, and other related molecular signaling pathways. These interactions are likely the key contributors to Bailing capsule’s efficacy in treating COPD (Figure 10(a)).

#### Molecular docking based on network pharmacology of Bailing capsules

In this section, we focus on the molecular docking interactions between Bailing capsule compounds and their target proteins. The top five critical targets identified from the network pharmacology study underwent semi-flexible docking with the key compounds of Bailing capsules.

Molecular Interaction Analysis: The binding affinity (energy) between the small molecular compounds and the target proteins was quantified, with binding energies less than zero indicating the potential binding. Lower binding energy values suggest a higher likelihood of binding. This analysis demonstrated that the compounds could effectively access the active sites of the target proteins. The most effectively docked molecules for each protein were presented in detail (Figure 10(b)).

Hydrogen Bonding Interactions: These interactions mean hydrogen bonds with specific amino acid residues associated with the target genes:

- HDAC2: Hydrogen bonds formed with GLU-109, ILE-80, and ASP-105, having lengths of 2.2 Å, 2.7 Å, and 2.4 Å, respectively.
- HIF1A: A hydrogen bond with TYR-276 at a length of 2.7 Å.
- NFKB1: Bonds with HIS-144, ASP-209, and LYS-147, with lengths ranging from 2.3 Å to 3.3 Å.
- PRKACA: Bonds with GLU-170, GLU-127, VAL-123, GLU-121, ranging from 2.1 Å to 2.9 Å.
- SRC: Bonds with GLN-528, LYS-203, GLU-178, and GLU-524, with lengths between 2.1 Å and 2.9 Å.

Additionally, these molecules formed strong hydrophobic interactions with surrounding amino acid residues. The detailed docking results, including binding energies and interaction patterns, are presented in Table 2.

**Table 2.** Molecular docking of *Cordyceps sinensis* mycobacteriosis.

Target point	PDB ID	Chemical compound	Binding energy (kcal/mol)
SRC	2SRC	Cordycepin	−7.1
HDAC2	6WBZ		−6.0
HIF1A	4H6J		−5.0
NFKB1	1SVC		−5.5
PRKACA	4WBB		−7.7

## Discussion

Today, COPD remains an incurable condition, with existing treatments aimed at managing symptoms, reducing exacerbation frequency, and improving exercise tolerance and overall health. However, these therapies do not arrest the long-term decline in lung function or overall patient health. To date, no pharmacotherapy has been proven to reverse lung function deterioration or cure COPD. However, evidence from the applications of TCM in the treatment of COPD indicates a significant improvement in its clinical symptoms, including cough, sputum production, wheezing, and shortness of breath in patients of the treatment group, compared to the control group, showing notable statistical significance (*p*** ***<*** **0.05). Secondary symptoms, including weakness, chills, and lumbar and knee pain, also improved considerably compared to the control group and showed statistical significance (*p*** ***<*** **0.05) (Wen 2018). Three studies showed no significant adverse reactions between the two groups (Hao et al. 2021; Xing et al. 2021, 2013). By contrast, one found that the adverse reactions improved with the use of Bailing capsules and conventional Western medicine (Wang et al. 2021). FEV1 is a critical indicator of individual-level lung function and is predictive of clinical outcomes, including mortality and the duration of hospitalization. Simultaneously, it is also considered in evaluating non-pharmacological COPD treatments, such as lung volume reduction or transplantation (Duong et al. 2019; Global Initiative for Chronic Obstructive Lung Disease (GOLD) 2023). Unfortunately, FEV1 is not a direct determinant of treatment due to the individual variances and is less critical than before, as it is not always associated with clinical symptoms impacting patients’ quality of life and the frequency of exacerbation (Van der Molen et al. 2013). It is now acknowledged that not all airway diseases result in abnormal spirometry findings (Rodriguez-Roisin et al. 2017). In our study, the FEV1 and FEV1/FVC ratio improved significantly in the group treated with Bailing capsule and conventional Western medicine, suggesting that Bailing capsule can notably enhance airway function, confirming its therapeutic efficacy. However, significant heterogeneity was also observed in these findings (*p*** **<** **0.00001, *I*^2^ = 92%), which may stem from differences in the duration of COPD and baseline lung function (FEV1 and FEV1/FVC) among patients. Patients with a longer disease duration and poorer baseline lung function showed more significant improvements with Bailing capsule treatment, whereas those with a shorter disease duration and better baseline lung function showed less improvement. These factors may impact the study results and will need to be explored further in future research to validate these sources of heterogeneity.

6MWT is a crucial tool for assessing the maximum exercise capacity of patients with COPD. In this test, the patient is requested to walk at their own pace as far as possible for 6 min. Herein, the primary outcome measure is a 6-min walk distance, which closely relates to the functional state daily and effectively reflects the patient’s exercise capacity and overall health condition. In healthy individuals, 6MWD is a robust predictor of peak oxygen consumption (VO2 max) and physical fitness, and is significantly correlated with mortality rates and the risk of acute exacerbation in COPD patients. Benzo’s study highlighted that a key predictor of one-year mortality in COPD patients was a reduction in 6MWD by 50 m or a decline in walking speed by 0.14 m/s from a six-month earlier assessment. Additionally, a decrease in the heart rate of less than 14 beats per min after a 1 min rest post-6MWT was found to be indicative of a higher risk of COPD exacerbation within a year (DePew et al. 2013). Exercise-induced desaturation (EID) during the 6MWT, defined as a drop in SaO_2_ of more than 4% post-exercise or a minimum SaO_2_ of less than 88%, is common in COPD patients. Those exhibiting EID after 1 min of walking are regarded as at risk for daily life hypoxia and have an elevated risk of developing respiratory failure over five years (Koopman et al. 2019). A 6MWD of fewer than 350 m indicates reduced exercise tolerance, which is also a risk factor for EID development. Other risk factors for EID include FEV1 as a percentage of the predicted value, pulmonary function classification, and SGRQ activity limitation score (Perez et al. 2019; Chang et al. 2020). Our meta-analysis revealed that the 6MWT in patients treated with Bailing capsules and the conventional treatment were significantly higher than those in the control group. This improvement demonstrates the therapeutic effectiveness of Bailing capsules, underscoring their clear efficacy in enhancing exercise tolerance in COPD patients.

Reducing acute exacerbations of COPD is a key objective of long-term maintenance treatment for COPD. Traditionally, the treatment of COPD has relied on the combination of anti-inflammatory drugs (e.g., inhaled corticosteroids (ICS)) and bronchodilators [e.g., long-acting β-agonists (LABA), and long-acting muscarinic antagonists (LAMA)]. However, the effectiveness of ICS in treating COPD with airway inflammation is still debated. Recent studies have suggested that the newer LABA+LAMA combination surpasses the ICS+LABA regimen in improving clinically important deterioration (CID) (Zhong et al. 2015; Wedzicha et al. 2016). Nevertheless, some study outcomes remain contentious. In severe, persistent COPD managed with ICS and salmeterol (or tiotropium bromide), the risk of moderate-to-severe exacerbations showed no significant difference when ICS was discontinued. However, a marked acceleration in lung function decline was observed after prolonged cessation of ICS therapy. Triple therapy (ICS+LABA+LAMA) may offer greater efficacy in reducing acute exacerbations of moderate-to-severe COPD compared to dual therapy (ICS/LABA) (Lipson et al. 2017). In this study, the analysis of Bailing capsules in conjunction with standard therapeutic agents in stable COPD revealed that Bailing-based combination therapy effectively improved lung function and exercise tolerance in the COPD population. Additionally, it reduced the frequency of acute exacerbations within one year, providing increased clinical benefits.

SGRQ is a standardized tool used to assess health-related quality of life in patients with COPD. Like lung function, quality of life is a crucial standard for evaluating the disease severity of COPD. The major influencing factors include smoking, elevated BMI, high cholesterol, depression or anxiety, air pollution, malnutrition, and comorbid conditions (Shahid et al. 2024). A study revealed that climate rehabilitation therapy may significantly improve both the physical and psychological states of COPD patients, uncovering key predictors of improved quality of life (Kubincová et al. 2023). Other research emphasized the impact of diet on COPD patients’ quality of life, identifying specific components, including protein, omega-3 polyunsaturated fatty acids, and vegetables that positively affect patient health (Fekete et al. 2023). In this study, an analysis of combining Bailing capsules with the standard treatment in stable COPD showed that this combination therapy could effectively improve the quality of life of patients with COPD.

The current expert consensus on immunomodulation therapy for COPD, informed by relevant research and global guidelines, has significantly influenced the clinical approach to the treatment of COPD in China. Current guidelines recognize that agents such as bacterial lysate products, phosphodiesterase inhibitors, and macrolides can modulate COPD by enhancing immune function and activating immune cells. Specifically, Bailing capsules have been shown to improve COPD symptoms via its immunomodulatory effects. Various studies have confirmed the role of Bailing capsules in regulating and enhancing immune function in stable COPD. Looking to the future, mesenchymal stem cells (MSCs) are emerging as a promising option for the treatment of COPD, particularly in repairing damaged lung tissues (Liu et al. 2016). MSCs not only possess anti-inflammatory and immune-modulating properties but also potentially reduce airway inflammation and emphysema. Research on human adipose-derived mesenchymal stem cells (hADSCs) suggests that they can differentiate into alveolar epithelial cells, impacting the progression of COPD. Another study on the autologous infusion of MSCs in COPD patients demonstrated its safety and feasibility (Mohammadipoor et al. 2018; Cho et al. 2019). It is important to note, however, that while these studies highlight the efficacy of MSCs in controlling COPD symptoms and enhancing patient quality of life, they do not suggest a cure for COPD.

Recent research on the pathogenesis of COPD has highlighted the significance of cellular processes such as proliferation, apoptosis, and autophagy, emphasizing the importance of autophagy in the progression of COPD. The HIF1A gene, which is associated with autophagy, is often upregulated in COPD patients, indicating its potential role in the disease’s development (Agusti et al. 2011; Doyle et al. 2013; Roche et al. 2013; Tsiligianni et al. 2016). Additionally, the SRC gene has been identified as a crucial factor in activating inflammatory mediators in COPD. As a key risk factor for COPD, smoking may activate genes that lead to airway inflammation. There is also evidence suggesting the involvement of the SRC gene in the activation of signaling cascades linked to tumorigenesis (Miravitlles et al. 2014). Another significant pathway related to COPD is the NF-κB1 signaling pathway, which is central to the inflammatory response. Studies have indicated the elevated expression of related genes in COPD, indicative of associations with the characteristics of the airway inflammatory response (Lange et al. 2014). Targeted pharmacotherapy that can modulate the expression of these genes could inhibit autophagy, thereby reducing airway inflammation. In summary, these approaches could contribute to alleviating COPD symptoms and managing the disease effectively and may in the near future provide helpful insights for the clinical treatment of COPD.

The network pharmacology analysis of Bailing capsules identified 122 common targets associated with COPD. Topological analysis of the protein interaction network revealed that SRC, HIF1A, NFKB1, HDAC2, and PRKACA could be the core targets of Bailing capsules, potentially improving lung function, enhancing exercise endurance, and reducing acute exacerbations of COPD. These findings align with previous research and reinforce the therapeutic potential of Bailing capsules in treating COPD. Predictive analysis of drug-disease targets also unveiled other related genes and molecular pathways involved in COPD regulation and treatment, such as CXCR4, PDGFRB, and PARP1 genes, along with cellular chemokines, tyrosine kinase receptors, and other molecular signaling pathways. Molecular docking studies are crucial for determining the drug action mode and binding affinity, significantly impacting drug efficacy and informing drug design. Understanding and optimizing the molecular structure of Bailing capsules can enhance drug-target binding, thereby improving therapeutic efficacy. The molecular docking results in this study emphasized the relationship between amino acid sites and drug binding strength. To enhance drug action, amino acids with weaker binding can be strategically modified, potentially boosting drug efficiency and clinical effectiveness. Deepening our understanding through drug networks and pharmacogenomic studies will continuously enrich our knowledge of the action mechanism underlying the action of Bailing capsules in the treatment of COPD. Exploring molecular targets and pathways offers insights into the mechanism, mode of action, and characteristics of Bailing capsules in COPD therapy. This molecular interaction research paves the way for tailored clinical drug applications and may become a guiding principle in future research directions for Bailing capsules. Such advancements are expected to lead to more targeted and effective clinical drug regimens, thereby promoting drug development for the clinical treatment of COPD.

Our meta-analysis results show that Bailing capsules combined with the conventional treatment significantly improve lung function and exercise endurance, reduce the frequency of acute exacerbations within one year, and thus significantly enhance the quality of life for patients. These results have important implications for clinical practice and treatment guidelines, providing an effective therapeutic option for the management of stable COPD. Notably, the network pharmacology and molecular docking studies could reveal the potential mechanisms of Bailing capsules, aiding in the formulation of more precise treatment strategies and possibly guiding the development and application of future COPD treatments. The results of this study provide a basis for further clinical research and help optimize treatment regimens for COPD patients, thereby improving their overall health and quality of life. However, further research is needed to comprehensively explore its mechanisms of action and potential in clinical applications. Therefore, future studies should focus on verifying the main active ingredients of Bailing capsules and their interactions with core targets through cell and animal model experiments, as well as conducting large-scale, multi-center, randomized controlled clinical trials to comprehensively evaluate potential clinical applications.

## Conclusions

The meta-analysis of various clinical studies indicated that Bailing capsules can significantly enhance lung function and exercise capacity during the stable phase of COPD. They were also found to play a crucial role in reducing acute exacerbations. Network pharmacology analysis provided deeper insights into the potential interaction targets of Bailing capsules, while molecular docking studies elucidated the binding characteristics of drug molecules to target proteins, affirming the active components of the medication. This comprehensive approach is instrumental in advancing the screening and design of therapeutic drugs for COPD, potentially augmenting their efficacy and clinical benefits for a broader patient population. However, further research is needed to verify these results and achieve better clinical practice outcomes.

## Data Availability

The datasets used and/or analyzed are available from the corresponding author upon reasonable request.
